# MolDy: molecular dynamics simulation made easy

**DOI:** 10.1093/bioinformatics/btae313

**Published:** 2024-06-12

**Authors:** Mohd Imran Khan, Sheetal Pathania, Mohammed W Al-Rabia, Abdul S Ethayathulla, Mohammad Imran Khan, Khaled S Allemailem, Mohd Azam, Gururao Hariprasad, Mohammad Azhar Imran

**Affiliations:** Division of Bioinformatics, AIBR Artificial Intelligence and Biochemical Research Pvt. Ltd., New Delhi 110076, India; Division of Bioinformatics, AIBR Artificial Intelligence and Biochemical Research Pvt. Ltd., New Delhi 110076, India; Department of Clinical Microbiology and Immunology, Faculty of Medicine, King Abdul Aziz University, Jeddah 21589, Saudi Arabia; Department of Clinical and Molecular Microbiology Laboratory, King Abdulaziz University Hospital, Jeddah 21589, Saudi Arabia; Department of Biophysics, All India Institute of Medical Sciences, New Delhi 110029, India; Research Center, King Faisal Specialist Hospital and Research Center, Jeddah 21589, Saudi Arabia; Department of Medical Laboratories, College of Applied Medical Sciences, Qassim University, Buraydah 51452, Saudi Arabia; Department of Medical Laboratories, College of Applied Medical Sciences, Qassim University, Buraydah 51452, Saudi Arabia; Department of Biophysics, All India Institute of Medical Sciences, New Delhi 110029, India; Division of Bioinformatics, AIBR Artificial Intelligence and Biochemical Research Pvt. Ltd., New Delhi 110076, India

## Abstract

**Motivation:**

Molecular dynamics (MD) is a computational experiment that is crucial for understanding the structure of biological macro and micro molecules, their folding, and the inter-molecular interactions. Accurate knowledge of these structural features is the cornerstone in drug development and elucidating macromolecules functions. The open-source GROMACS biomolecular MD simulation program is recognized as a reliable and frequently used simulation program for its precision. However, the user requires expertise, and scripting skills to carrying out MD simulations.

**Results:**

We have developed an end-to-end interactive MD simulation application, MolDy for Gromacs. This front-end application provides a customizable user interface integrated with the Python and Perl-based logical backend connecting the Linux shell and Gromacs software. The tool performs analysis and provides the user with simulation trajectories and graphical representations of relevant biophysical parameters. The advantages of MolDy are (i) user-friendly, does not requiring the researcher to have prior knowledge of Linux; (ii) easy installation by a single command; (iii) freely available for academic research; (iv) can run with minimum configuration of operating systems; (v) has valid default prefilled parameters for beginners, and at the same time provides scope for modifications for expert users.

**Availability and implementation:**

MolDy is available freely as compressed source code files with user manual for installation and operation on GitHub: https://github.com/AIBResearchMolDy/Moldyv01.git and on https://aibresearch.com/innovations.

## 1 Introduction

In biomedical research, understanding the protein dynamic states, their interactions with other proteins, peptides and small molecules and their kinetic mechanism are critical for structure-based drug discovery (Batool *et al.* 2019). Experimentally determining the intrinsic dynamics of ligand binding is challenging and time consuming due to its volatile nature (Buch *et al.* 2011). The focus has generally shifted on computational prediction of characteristic transition states of a biomacromolecule systems by mimicking its natural environment of solvents for understanding, controlling, and reengineering the process of finding the lowest energy states and studying the dynamic behavior of atoms and molecules involved (Bernetti *et al.* 2017, Rácz *et al.* 2022). Molecular dynamics (MD) facilitates the accurate evaluation of macromolecular behavior, qualitative binding affinity and the kinetic parameters where performing experimental studies is difficult, and expensive (Perez *et al.* 2014, Pantsar and Poso 2018). By evaluating the thermodynamic characteristics of the solvents at protein-binding sites using MD simulations one can understand allosteric mechanisms that can be used for designing drugs (Torres *et al.* 2019). Most recently Tesei and group utilized MD simulation to develop an efficient model to identify the relationship between sequence, conservation, conformational ensembles, biological function and disease variants of the intrinsically modified regions at proteome scale (Tesei *et al.* 2024).

Gromacs is a widely used freely available software conventionally used for biomolecular simulations. It supports advanced parallel computational algorithms for enormous calculations and thus features multithreading, GPU acceleration, domain decomposition and ensemble parallelization during the process. Gromacs allows the user to customize the defined parameters as per their experiments (Pronk *et al.* 2013, Abraham *et al.* 2015). However, the intricacy of setting up, executing, and assessing MD simulations for various systems sometimes poses a challenge for beginners, in terms of time and analysis hence requires expertise (Kagami *et al.* 2020, Bayarri *et al.* 2022, Maity and Pal 2022). To overcome these lacunae, we have developed an automated user-friendly Gromacs GUI interface, MolDy, which performs complex MD simulation easily, smoothly and efficiently.

## 2 Materials, methods, and operation

### 2.1 Platform development

**Figure 1.**
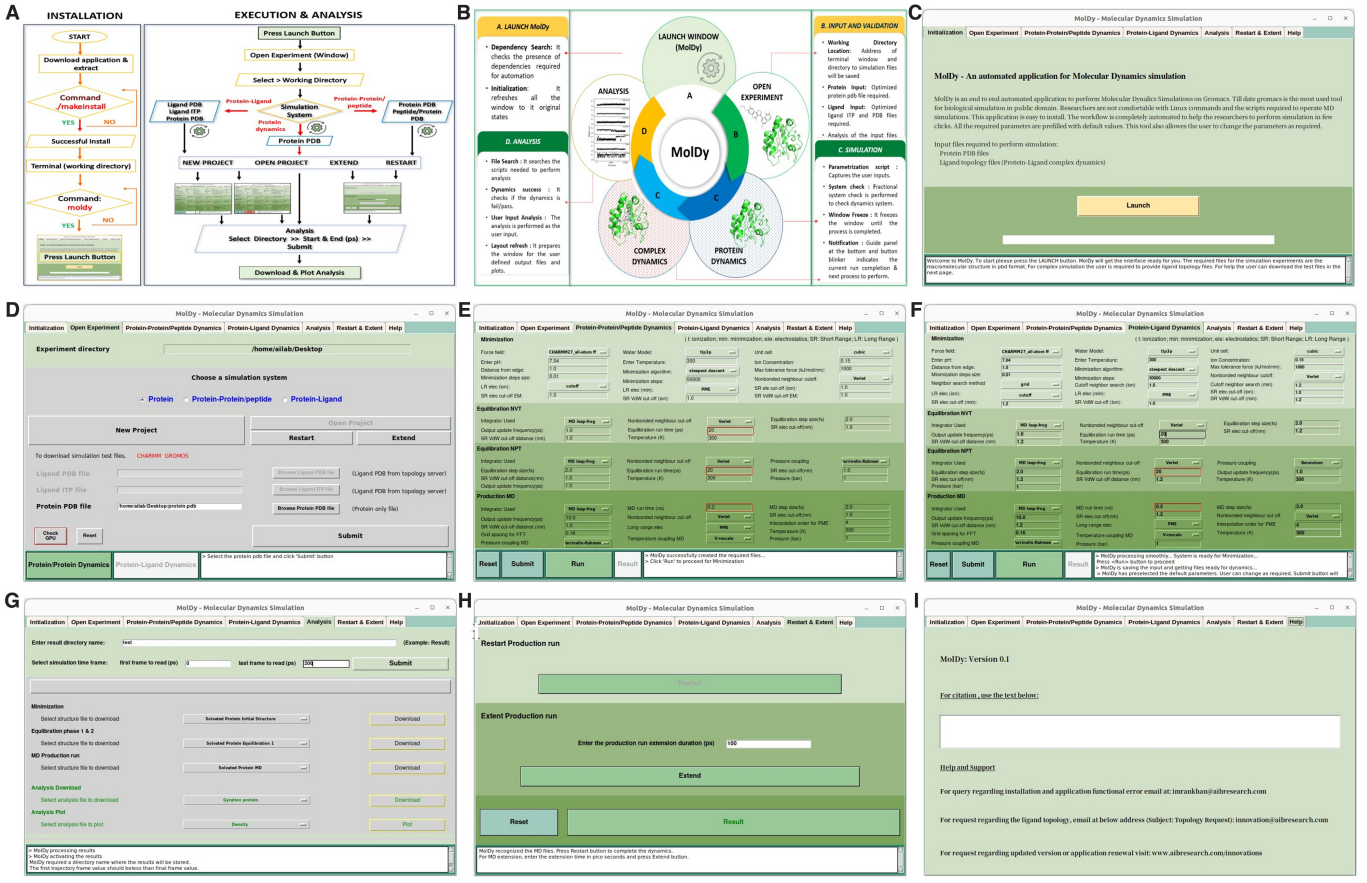
Installation, process flowchart depicting entire process of the application and snapshots of MolDy application windows. (A) A brief diagrammatic description of the MolDy architecture; (B) installation and process followed by MolDy to perform MD simulation; (C) diagrammatic representation of MolDy front end Initialization window; (D) open experiment window; (E) dynamics window of protein-based simulation experiments; (F) dynamics window of protein–ligand complex-based simulation experiments; (G) analysis window; (H) restart and extend window; (I) help window.

MolDy is developed as an integrative Linux-based software pipeline as a GUI of Gromacs. The user interactive front-end and back-end codes have been linked to a logical middle layer script that maintains communications and decides a set of codes that is to be triggered as per the requirements. Gromacs modules based on the UNIX shell have been used for execution and analysis. All the codes were written in PERL, Python, Python-Tk and C-languages. The package includes scripts for installation, execution, and analysis of MD simulation. MolDy consists of 6 self-explanatory tabs/windows (Fig. 1A).

### 2.2 Comparison of MolDy with other GROMACS-based GUI

Operational abilities of MolDy were compared with other GROMACS-based GUIs that have been developed in the recent past.

## 3 Functioning of MoldDy

### 3.1 Installation

MolDy is a single command installation framework (./makeinstall). However, to run MolDy with GPUs, a preinstalled functional GPU is essential.

### 3.2 Operation

The interface is designed to help the user navigate through the experimental workflow (Fig. 1B). To launch MolDy, the user simply need to call the moldy command in a terminal window from the working directory. It opens a front-end interactive application which informs the user of operations, errors and warnings through popup messages. The overall MD simulation process is segregated into seven separate modules depicted as windows.

#### 3.2.1 Initialization window

MolDy software starts with the initialization window with a launch button (Fig. 1C). At this step, the application verifies the presence of dependencies and files required for running MD simulations by switching to the experiment window mode.

#### 3.2.2 Open experiment window

The user has the options to select the MD simulations system based on the experimental requirement like (i) protein, (ii) protein–protein, or (iii) protein–ligand dynamics (Fig. 1D). The entire process is coded in a way to guide the user through the process in a sequential way. Once the simulation system type is selected, the user can choose options (i) New Project: to start as a fresh experiment, (ii) Open Project: to perform the analysis on the pre-existing MD experiments, (iii) Restart: to continue the pre-existing incomplete simulation experiment, and (iv) Extend: to continue the simulation period of the preexisting dynamics experiment. Based on the user input appropriate input file window will be activated. User is required to upload appropriate input pdb/itp files as per the experimental requirement which will initiate the corresponding dynamics protocol. White window at the bottom will display the functioning and guide the user throughout the application. Additionally, the window contains a button ‘Check GPU’ which will popup the information regarding the graphic card. Input example files for CHARMM and Gromos force field are provided. Upon clicking the text link (CHARMM or GROMOS) relevant test files will be downloaded into the experiment directory.

#### 3.2.3 Protein dynamics window

The protein dynamics window contains >50 pre-filled minimization, equilibration and MD production run parameters (Fig. 1E). These parameters are editable for users to change as per their experimental needs. The reset button will shuffle the window with the default parameters. The Run button will start the simulation process. Upon completion of simulation, it will confirm the completion of MD simulation and subsequently the analysis button will be activated. It will also check for the successful job and transfer the user to the Analysis window.

#### 3.2.4 Protein–ligand complex dynamics window

The window appearance and workflow are very similar to the protein–protein dynamics with some changes in parameter values as shown in Fig. 1F. The Submit, Run, and Analysis buttons will work the same way.

#### 3.2.5 Analysis and result generation window

The analysis window will help the user to create different analyses according to their research requirements (Fig. 1G). The user is required to provide the start and end time points to be considered for MD analysis. Submit button followed by the Process result will update the relevant options in the dropdown list. The user can select the desired option and click download or plot button to access the result.

#### 3.2.6 Restart and extend

In instances where the simulation process is discontinued, the restart and extend window will help the user to restart from the nearest checkpoint registered by the Gromacs (Fig. 1H). This function is enabled by checking the presence of an incomplete production MD run and restart the simulation task again.

#### 3.2.7 Help window

The help window contains information about application (Fig. 1I). Apart from this, a user can access support regarding the application installation, operation and topology generation. MolDy is committed for future updates to help the user with subsequent upgrades associated with Gromacs and Ubuntu.

#### 3.2.8 Troubleshooting

MolDy operations are based on input structure and topology files for protein and complex dynamics simulation. Therefore, any inconsistency in the submitted files (.pdb and .itp) may lead to errors and warnings. The errors and their possible reasons along with their solutions are tabulated and can be accessed in Supplementary Table S2.

## 4. Results

### 4.1 Interface

The software package comprises a single compressed folder with all the required files necessary for the installation and operation. Installation is a single-step process that can be run from any location in the Ubuntu 2020 or higher systems. The process will check all the required dependencies and install them with the user’s consent. The reinstallation of the software is self-sufficient and without any prior requirements of purging the previously installed libraries. This process takes no more than a few minutes. The front end contains seven different compact windows which are script-free for the user as compared to other highly technical scripting-based approaches. The tool is also capable of performing analysis of the dynamics to provide the user with simulated structures, relevant plots and well-formatted biophysical parameters in publishable formats.

### 4.2 Comparison of MolDy with other GROMACS-based GUI

MolDy is the only interface with ‘One-step’ installation with no parallel dependency requirements reestablishing its user-friendly model. Additionally, the tool provides >50 simulation parameters selections that allows the user with an easy customization which are very limited in all other GUIs. A brief comparison can be found in Supplementary Table S1. These features are a major advantage that makes it a highly customizable application for advanced users.

## 5. Conclusion

MolDy is a highly user-friendly application and ‘click of a button’ approach for beginners along with parameter customizability options for advanced researchers in the fields of computational biology and structural bioinformatics. The program is a combination of a tailored front-end along with fully automated back end to perform simulations of protein and protein–ligand complexes. MolDy stands out as being the most advanced tool for MD simulations on Gromacs.

## 6. Future software updates

The authors are committed to regularly provide updates to maintain its workability and enhanced functionality.

## Supplementary Material

btae313_Supplementary_Data
