# Supplementary material for: MolDy: molecular dynamics simulation made easy
Source: Bioinformatics. 2024 Jun 12;40(6):btae313. doi: 10.1093/bioinformatics/btae313 (PMC11187490; doi:10.1093/bioinformatics/btae313)
Supplement: btae313_Supplementary_Data [file btae313_supplementary_data.docx]

**Supplementary data**

**Supplementary Table S1.** Comparison of MolDy with other Gromacs front-end applications

| **Gromacs applications** | **Year** | **One-step installation** | **Working Status** | **Gromacs v2020, or higher** | **Easy front-end customizability parameters (>50)** | **Preinstalled dependencies** |
| --- | --- | --- | --- | --- | --- | --- |
| MolDy | 2023 | ✓ | ✓ | ✓ | ✓ | Not Required |
| YAMACS (Sarkar et al., 2022) | 2022 | 🗶 | ✓ | ✓ | 🗶 | Required |
| Dynamics Pymol plugin (Makarewicz et al., 2013) | 2013 | 🗶 | 🗶 | 🗶 | 🗶 | Required |
| MDWeb (Hospital et al., 2012) | 2012 | 🗶 | 🗶 | 🗶 | 🗶 | Required |
| Gromita (Sellis et al., 2009) | 2009 | 🗶 | 🗶 | 🗶 | 🗶 | Required |
| GUIMACS (Kota et al., 2007) | 2007 | 🗶 | 🗶 | 🗶 | 🗶 | Required |

**Supplementary Table S2.** Possible errors and troubleshooting in MolDy.

| **MolDy windows** | **Errors** | **Probable reasons** | **Solutions** |
| --- | --- | --- | --- |
| **Initialization** | Check the internet connection | No internet connectivity. | Restart the application |
| **Open Experiment** | Working Directory error | MolDy started from a different directory | Restart MolDy using the terminal window from the working directory |
|  | Protein PDB Warning | Input protein PDB contains atoms other than proteins | Click ‘Yes’ on the warning to let the MolDy remove all other Heteroatoms. |
|  | Multi-Ligand PDB warning | Ligand PDB contains multiple structures | Rectify ligand PDB file |
|  | Ligand PDB warning | Ligand molecule nomenclature error in the PDB | Rectify ligand PDB file |
|  | Ligand topology warning | Ligand topology detail incorrect | Rectify ligand ITP file |
|  | Ligand topology warning | Ligand PDB and topology files don’t correspond. | Rectify ligand ITP file. Probably user must use PDB and ITP files from the same source. |
| **Protein Dynamics** | Input files and parameters error | Supplied protein file is not complete or it has errors.  Input dynamics parameters are not correct or allowed | Corrected protein for gaps, missing atoms, and terminus atoms. Correct input dynamics parameters |
| **Protein-Ligand Dynamics** | Input files and parameters error | Supplied protein file is not complete or it has errors.  Input dynamics parameters are not correct or allowed | Corrected protein for gaps, missing atoms, and terminus atoms. Correct input dynamics parameters |
| **Error while running Equilibration or MD** | Errors with Codes | Dynamic system blown, Segmentation fault, GPU error | Try to restart or repeat |
